# A rare case report of hemolysis in a newborn: hereditary elliptocytosis

**DOI:** 10.3389/fped.2024.1485318

**Published:** 2024-10-22

**Authors:** Shouliang Jiang, Ruifeng Lu, Jun Tang

**Affiliations:** Key Laboratory of Obstetric & Gynecologic and Pediatric Diseases and Birth Defects of Ministry of Education, Department of Pediatrics, West China Second University Hospital, Sichuan University, Chengdu, China

**Keywords:** hereditary Elliptocytosis, SPTA1, neonate, hemolysis, hemolytic jaundice

## Abstract

**Introduction:**

Hereditary Elliptocytosis (HE) comprises clinically and genetically heterogeneous red cell membranopathies resulting from defects in the horizontal linkage between red blood cell (RBC) membrane and cytoskeletal proteins, which affect mechanical stability and deformability, thereby reducing RBC lifespan. The principal defect in HE is due to dysfunction or deficiency of RBC cytoskeletal proteins.

**Case description:**

This study reported a case of severe hemolysis occurring within one day after birth in a term newborn. High-throughput sequencing was used to characterize the pathogenic gene variation in this child and to study the correlation between the identified variation and its corresponding phenotypic characteristics.

**Conclusion:**

HE is caused by monoallelic mutations, which justify the phenotypic heterogeneity observed in patients. Furthermore, molecular analysis using high-throughput sequencing enables diagnosis in disorders with highly variable heterogeneity. HE can also present with severe hemolysis during the neonatal period.

## Introduction

Hemolytic disease of the newborn typically refers to hemolysis caused by Rh or ABO incompatibility (1, 2), but a small proportion of cases are attributed to abnormal red blood cell morphology, erythrocyte membrane defects, or erythrocyte enzyme deficiencies. In severe cases, blood exchange therapy may be required and can be life-threatening.

Hereditary Elliptocytosis (HE) is a rare form of hemolysis with a global distribution. The prevalence of HE in Europe and the United States is approximately 0.03% to 0.05%, while in malaria-endemic regions such as Africa, it can reach 0.6%–1.6% (3, 4). HE is characterized by elliptic erythrocytes in peripheral blood, and its diagnosis primarily relies on medical history, peripheral blood smear, and sodium dodecyl sulfate polyacrylamide gel electrophoresis. Children with onset in the neonatal period have a relatively poor prognosis, possibly due to bilirubinemia from red blood cell destruction leading to bilirubin encephalopathy. Splenectomy is generally recommended for children who require prolonged transfusion therapy and children over 6 years who do not respond well to prolonged medical treatment (5, 6).

However, due to the significant clinical heterogeneity among patients, some cases may be missed with these diagnostic methods alone. The advent of high-throughput sequencing has enabled more accurate diagnosis through comprehensive gene examinations and has facilitated the study of disease mechanisms at the molecular level. This article presents a case of a rare HE patient with neonatal hemolysis, characterized by a positive SPTA1 gene mutation, along with relevant parental genetic information (3, 7).

## Case description

The child was admitted to the hospital with the chief complaint of “yellowing of the skin for 4 h,” at 9 h and 18 min of age. Four hours prior to admission, the parents had noticed yellowing of the child's face and skin. Laboratory results showed total bilirubin 271 μmol/L, and indirect bilirubin 256.9 μmol/L. The child exhibited no lethargy, agitation, crying, convulsions, reduced feeding, or decreased activity. Urine and feces were passed normally, and no abnormalities were noted in appearance. The child was receiving postnatal mixed feeding at 10 ml per feeding, every 3 h.

Personal History: G3P2, born at 38 + 6 weeks, with an uneventful delivery. There was no intrauterine distress, premature rupture of membranes, or umbilical cord wrapping around the neck. Amniotic fluid was clear and bright, at 350 ml. Birth weight was 2,600 g, and Apgar scores were all 10 at 1–5–10 min.

### Family history

The child has a 3-year-old sister with no specific clinical presentation.

### Physical examination

Temperature 36.5°C, pulse 134 beats/min, respiration 50 breaths/min, blood pressure 73/22 mmHg, admission weight 2.58 kg. Yellowing of the skin was observed on the face, neck, and trunk. Liver and spleen were not enlarged on palpation. Examination of the heart, lungs, abdomen, and neurological system revealed no abnormalities. Urine and feces were normal in color.

### Laboratory results

**Figure 1:**
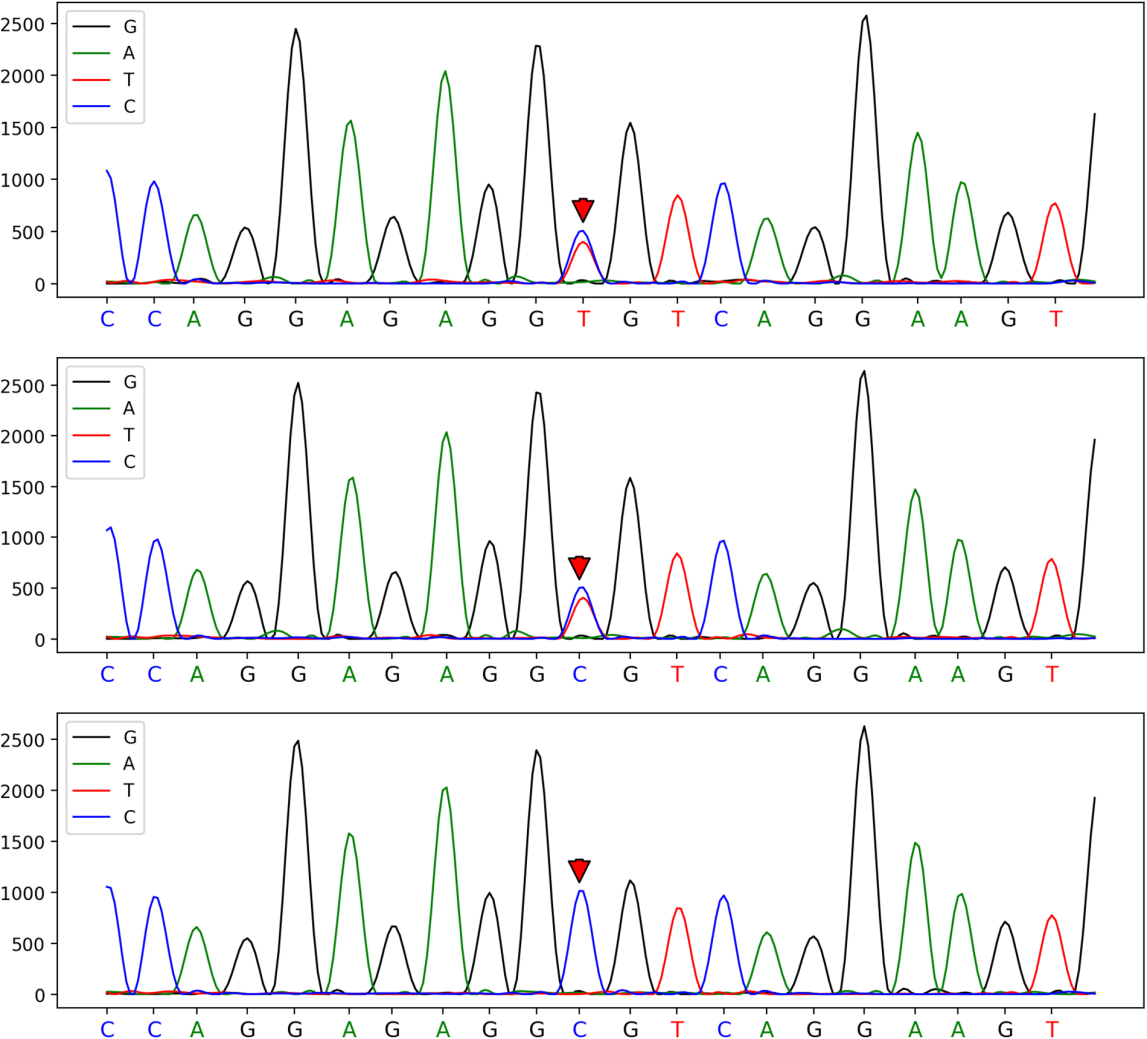
A heterozygous mutation at the c.82C>T mutation site of the patient, which was confirmed by one-generation sequencing to have originated from his father's chromosome position: chr1:158655080.

Routine haematology and Trends in bilirubin are detailed in Table 1 and Table 2. The direct and indirect anti-human globulin tests, blood group antibody screen, hemoglobin electrophoresis, G6PD test, H inclusion bodies, and blood and urine genetic metabolism screens were negative. The pyruvate kinase test was not performed due to lack of available equipment. Whole exome sequencing identified a mutation in the SPTA1 gene (c.82C>T) at chromosome position chr1:158655080, as shown in Figure 1 and Table 3 one week later after admission.

**Table 1:** Routine haematology shows trends in haemoglobin.

Date	WBC (10^9^/L)	RBC (10^12^/L)	Hb (g/L)	HCT (%)	MCV (fl)	MCH (pg)	RDW-CV (%)	RDW-SD (fl)	RET%	RET# (10^12^/L)	PLT (10^9^/L)
2024.03.20 17:52	32.2	5.52	142	43.7	79.2	25.7	41.2	/	12.67	0.6626	377
3.20 21:12	24.6	4.36	112	36.1	82.8	25.7	41.1	/	11.07	0.4827	355
3.21 01:46	16.5	3.02	79	25	82.8	26.2	39.3	/	/	/	267
3.21 04:38	6.1	6.08	172	51.6	84.9	28.3	17.7	50.2	/	/	82
3.21 09:31	9.2	6.27	175	50.5	80.5	27.9	18.2	48.4	/	/	71
3.21 16:42	3.5	4.86	150	42.6	87.7	30.9	13.7	43.4	/	/	50
3.22 06:21	4.3	4.40	138	38.4	87.3	31.4	13.7	42.8	/	/	41
3.22 15:44	5.0	5.31	158	46.1	86.8	29.8	14.4	44.4	/	/	43
3.23 15:31	5.5	4.76	148	42.9	90.1	31.1	14.7	48.6	/	/	47
3.24 07:08	7.1	4.42	135	40.0	90.5	30.5	14.7	48.7	/	/	50
3.26 15:13	14.2	4.23	131	38.7	91.5	31.0	14.5	48.7	/	/	144
3.28 10:02	11.7	4.18	128	39.6	94.7	30.6	14.1	49.5	/	/	263
4.01 15:29	9.3	3.63	110	34.2	94.2	30.3	13.6	47.7	/	/	414
4.03 04:26	9.1	3.24	99	30.3	93.5	30.3	13.5	46.4	/	/	404

**Table 2:** Trends in bilirubin (including total and indirect bilirubin).

Date	TB (μmol/L)	IDIL (μmol/L)
2024.03.20 18:58	271	256.9
3.20 23:05	310.8	283.6
3.21 03:39	298	274
3.21 05:51	222.2	209.4
3.21 11:51	269.5	237.9
3.21 17:41	172.4	162.8
3.21 23:51	226.5	205.9
3.22 07:28	208.0	184.4
3.22 17:18	192.7	173.2
3.23 17:32	169.2	153.8
3.24 08:13	136.7	119.6
3.26 16:46	87.7	64.7
3.28 11:56	74.3	58.0
4.01 17:58	31.8	19.8

**Table 3:** A mutation in the SPTA1 gene was found on chromosome chr1:158655080 (c.82C > T).

Second generation sequencing								
Site	Gene	Transcript exon	Nucleotide change. Amino acid change. Chromosome location	HGMD report	MAF	ACMG classify	Genetically related diseases	Proband
1	SPTA1	NM_003126 exon2	c.82C>T p.R28C chr1:158655080	Elliptocytosis	No included	May cause disease	Hereditary febrile polycythemia (266140). elliptocytosis type 2 (130600). spherocytosis type 3 (270970).	Heterozygosis

### Treatment

At admission, bilirubin levels met the criteria for blood exchange therapy. Following admission, there was a progressive decrease in hemoglobin (from 142 g/L to 112 g/L, as shown in Table 1) and a progressive increase in bilirubin (total bilirubin from 271 μmol/L to 310.8 μmol/L, as shown in Table 2). Consequently, the newborn underwent the first blood exchange. After the initial transfusion, total bilirubin decreased to 222.2 μmol/L. However, 8 h later, total bilirubin rose to 269.5 μmol/L, again meeting the criteria for blood exchange therapy. Therefore, the newborn received a second blood exchange therapy on the first day of life. After two blood exchanges, the child's blood bilirubin did not rebound significantly, although haemoglobin showed a slow decline. Additional treatments included intensive phototherapy, albumin infusion, and human immunoglobulin administration. Based on the child's history, physical examination, treatment and laboratory tests, the diagnosis of Hereditary Elliptocytosis was considered.

The child was followed up in the outpatient clinic after discharge from the hospital. The child's response to breastfeeding was acceptable, and the routine blood test showed a slight decrease in haemoglobin compared with the previous period, but the elevation of bilirubin was not obvious.

## Discussion

Hemolytic disease of the newborn that presents early in the postnatal period is often characterized by hemolysis due to blood group incompatibility, especially if the onset occurs within 24 h. Given this, the primary consideration upon the child's admission was whether the hemolysis was due to blood group incompatibility. However, this was ruled out as there were no risk factors for mother-child blood group incompatibility, including both ABO and non-ABO blood group incompatibility hemolytic diseases.

Next, we considered intravascular hemolysis due to red cell membrane abnormalities, red cell enzyme deficiencies, red cell structural abnormalities, and hemoglobinopathies. However, the child's test results, including hemoglobin electrophoresis, G6PD, and Heinz bodies (also known as denatured bead vesicles or Heinz microsomes), were all negative. No specific erythrocyte morphologic or structural abnormalities were detected, even upon early microscopic examination.

This was perplexing. Despite the initial emergency blood exchange treatment, hemolysis persisted and jaundice worsened (8). This led to the decision for a second blood exchange treatment. Concurrently, human albumin was infused to aid in the transport of indirect bilirubin. Notably, the ratio of total bilirubin to albumin was greater than or equal to 7.2. After two blood exchange treatments and blue light phototherapy, the child's bilirubin and hemoglobin levels stabilized within the normal range. Due to the impact of blood exchange on routine blood smear results, these were not clinically specific. However, genetic testing was performed using whole exome sequencing. This identified a clinically significant gene mutation: SPTA1 (c.82C>T) at chromosome position chr1:158655080, a heterozygous mutation of paternal origin.

SPTA1 is the most frequently mutated gene in hereditary elliptocytosis (HE), with mutations predominantly occurring in exon 2. Point mutations are the most common, and missense mutations are the most prevalent type. There appears to be no significant correlation between HE genotypes and clinical phenotypes. We hypothesized that the mutation in the c.82-c.83 region of exon 2 of the SPTA1 gene was responsible for the condition (9). Reports indicate that patients with mutations in exon 2 of the SPTA1 gene experience varying degrees of anemia, which may be due to incomplete exclusion of the mutated locus, where one allele may be silenced and not expressed (10, 11). The variability in the number of *α* chains encoded by the SPTA1 gene among individuals can lead to different clinical manifestations, even with the same gene mutation. This variability may explain why our patient experienced severe hemolysis within one day after birth.

The SPTA1 gene is located on 1q23.1 and consists of 52 exons. Alpha-hemoglobin is a crucial component of the erythrocyte cytoskeleton. Mutations in this gene can disrupt the horizontal connections within the erythrocyte skeleton, leading to decreased erythrocyte stability and deformability, and resulting in hemolysis (12). Missense mutations in the NH2 terminal region of alpha-hemoglobin are among the most common defects in Hereditary Elliptocytosis (HE) and hereditary pyropoikilocytosis (HPP) (13, 14).

HE is typically caused by monoallelic mutations, while the more severe HPP is usually caused by biallelic (homozygous or compound heterozygous) mutations, which accounts for the phenotypic differences observed in patients. In this study, high-throughput sequencing offered a rapid and efficient method for diagnosing HE and HPP, especially in severe cases where the RBC phenotype could not be assessed. Accurate diagnosis in such heterogeneous disorders requires combining clinical data with family studies to understand the significance of new genetic variants in the pathophysiology of RBC cytoskeletal disorders. This approach helps elucidate the genotypic-phenotypic correlation and provides valuable genetic counseling for patients and their families.

High-throughput sequencing involves randomly interrupting the patient's DNA into numerous small fragments (250–300 bp) by certain methods, after which these fragments are enriched by building libraries, and the built libraries are put into a sequencer to be sequenced. The results are then verified against the parents’ genes by generational sequencing (15). At the time of discharge, the child's family expressed satisfaction with the treatment received.

## Data Availability

The authors acknowledge that the data presented in this study must be deposited and made publicly available in an acceptable repository, prior to publication. Frontiers cannot accept a manuscript that does not adhere to our open data policies.
