# Supplementary material for: A rare case report of hemolysis in a newborn: hereditary elliptocytosis
Source: Front Pediatr. 2024 Oct 22;12:1485318. doi: 10.3389/fped.2024.1485318 (PMC11534593; doi:10.3389/fped.2024.1485318)
Supplement: Supplementary file 1 [file Datasheet1.pdf]

CARE case report guidelines

CARE Checklist of information to include when writing a case report

(cc) BY-NC-ND

Topic	Item	Checklist item description	Reported on Line
Title	1	The diagnosis or intervention of primary focus followed by the words "case report"	1
Key Words	2	2 to 5 key words that identify diagnoses or interventions in this case report, including "case report"	16
Abstract (no references)	3a	Introduction: What is unique about this case and what does it add to the scientific literature?	18-22
	3b	Main symptoms and/or important clinical findings	23-26
	3c	The main diagnoses, therapeutic interventions, and outcomes	23-26
	3d	Conclusion—What is the main "take-away" lesson(s) from this case?	27-30
Introduction	4	One or two paragraphs summarizing why this case is unique (**may include references**)	32-45
Patient Information	5a	De-identified patient specific information.	48-49
	5b	Primary concerns and symptoms of the patient.	48-50
	5c	Medical, family, and psycho-social history including relevant genetic information	55-58
	5d	Relevant past interventions with outcomes	51-54
Clinical Findings	6	Describe significant physical examination (PE) and important clinical findings.	59-63
Timeline	7	Historical and current information from this episode of care organized as a timeline	NA
Diagnostic Assessment	8a	Diagnostic testing (such as PE, laboratory testing, imaging, surveys).	64-69
	8b	Diagnostic challenges (such as access to testing, financial, or cultural)	64-71
	8c	Diagnosis (including other diagnoses considered)	70-71
	8d	Prognosis (such as staging in oncology) where applicable	68-69
Therapeutic Intervention	9a	Types of therapeutic intervention (such as pharmacologic, surgical, preventive, self-care)	71-79
	9b	Administration of therapeutic intervention (such as dosage, strength, duration)	71-79
	9c	Changes in therapeutic intervention (with rationale)	71-79
Follow-up and Outcomes	10a	Clinician and patient-assessed outcomes (if available)	NA
	10b	Important follow-up diagnostic and other test results	80-82
	10c	Intervention adherence and tolerability (How was this assessed?)	80-82
	10d	Adverse and unanticipated events	80-82
Discussion	11a	A scientific discussion of the strengths AND limitations associated with this case report	122-129
	11b	Discussion of the relevant medical literature **with references**.	85-121
	11c	The scientific rationale for any conclusions (including assessment of possible causes)	122-129
	11d	The primary "take-away" lessons of this case report (without references) in a one paragraph conclusion	122-129
Patient Perspective	12	The patient should share their perspective in one to two paragraphs on the treatment(s) they received	130
Informed Consent	13	Did the patient give informed consent? Please provide if requested	**Yes** ☑ **No** ☐
